# Field-Programmable Gate Array (FPGA)-Based Lock-In Amplifier System with Signal Enhancement: A Comprehensive Review on the Design for Advanced Measurement Applications

**DOI:** 10.3390/s25020584

**Published:** 2025-01-20

**Authors:** Jose Alejandro Galaviz-Aguilar, Cesar Vargas-Rosales, Francisco Falcone, Carlos Aguilar-Avelar

**Affiliations:** 1 Tecnologico de Monterrey, School of Engineering and Science, Monterrey 64849, Mexico; galaviz@tec.mx (J.A.G.-A.); cvargas@tec.mx (C.V.-R.); francisco.falcone@tec.mx (F.F.); 2 Institute of Smart Cities, Universidad Pública de Navarra, Campus Arrosadía, E-31006 Pamplona, Spain; 3 Facultad de Ingeniería, Universidad Autónoma de Baja California, Mexicali 21280, Mexico

**Keywords:** additive white Gaussian noise (AWGN), lock-in amplifiers (LIAs), field-programmable gate array (FPGA), phase sensitive detector (PSD), spurious-free dynamic range (SFDR)

## Abstract

Lock-in amplifiers (LIAs) are critical tools in precision measurement, particularly for applications involving weak signals obscured by noise. Advances in signal processing algorithms and hardware synthesis have enabled accurate signal extraction, even in extremely noisy environments, making LIAs indispensable in sensor applications for healthcare, industry, and other services. For instance, the electrical impedance measurement of the human body, organs, tissues, and cells, known as bioelectrical impedance, is commonly used in biomedical and healthcare applications because it is non-invasive and relatively inexpensive. Also, due to its portability and miniaturization capabilities, it has great potential for the development of new point-of-care and portable testing devices. In this document, we highlight existing techniques for high-frequency resolution and precise phase detection in LIA reference signals from field-programmable gate array (FPGA) designs. A comprehensive review is presented under the key requirements and techniques for single- and dual-phase digital LIA architectures, where relevant insights are provided to address the LIAs’ digital precision in measurement system configurations. Furthermore, the document highlights a novel method to enhance the spurious-free dynamic range (SFDR), thereby advancing the precision and effectiveness of LIAs in complex measurement environments. Finally, we summarize the diverse applications of impedance measurement, highlighting the wide range of fields that can benefit from the design of high performance in modern measurement technologies.

## 1. Introduction

In many scientific and industrial applications, there is a need to measure or extract a signal with an amplitude that is much smaller than the noise component present in the environment [1,2,3]. The development in the design and manufacturing of sensors has led to a significant increase in their electronic sensitivity, such as that needed in integrated solutions based on lock-in amplifiers (LIAs), which is a rigorous yet practical framework for electronic measurements, considered fundamental for instrumentation, design applications, and characterization in modern physical environments. Field-programmable gate arrays (FPGAs) have emerged as indispensable tools in the design of advanced digital LIAs used in precision measurement applications. Their inherent flexibility, high-speed processing capabilities, and parallel architecture make FPGAs particularly well suited for the computational demands of digital signal processing and real-time analysis.

In addition to computational efficiency, FPGAs facilitate rapid prototyping and iterative design, which are essential for developing LIAs tailored to specific measurement requirements. For example, in biomedical applications, such as electrical impedance tomography or bioimpedance spectroscopy, the FPGA’s ability to handle high-frequency signals and maintain signal integrity across multiple channels is pivotal. Furthermore, the scalability of FPGA-based designs supports their integration into portable and cost-efficient measurement systems, expanding the applicability of LIAs in diverse fields. Another approach is the development of biomedical databases, electronic health records (EHRs), and public health, which have been enhanced not only by availability and traceability but also by the liquidity of heterogeneous healthcare data obtained from several environment sensors [4]. One can find applications of LIAs in every sector of industrial electronics, including the service industry, such as in telecommunications systems, electronics design, healthcare devices, the manufacturing of measurement equipment, and any application where the signal to be measured is several times smaller than the background noise [5,6,7]. Lock-in amplifier circuit integration, along with the design of sensor elements, has seen a reduction in cost and power consumption [8,9,10,11,12]. Digital LIAs (DLIAs) have become increasingly popular in many experimental configurations due to the combination of flexibility, cost, and performance [13]. Current research efforts on signal acquisition provide solid foundations for the development of embedded design, VLSI systems, and applications with a special emphasis on application-specific integrated circuit (ASIC) integration, for instance, to build smart and fully embedded EEG sensing systems [14,15].

This paper aims to provide an overview to comparatively analyze LIA architectures from the perspective of their design, implementation, and applications to give the reader a general point of view of the importance of a functional digital design. This work discusses the practicality of features of cost-efficient, portable, and digital customizable LIA systems. Hence, the role of lock-in amplifiers and the state of the art of the FPGA system design implementations are reviewed. The rest of this paper is organized as follows. Section 2 describes the fundamentals of LIA systems. Section 3 provides comprehensive and detailed review for the single-phase and dual-phase LIAs architectures and their basic operation. In Section 4, the LIAs digital approach from the perspective of critical design issues and a phase-dithering technique for signal generation enhancement are discussed. Section 4 provides a detailed implementation review of FPGA-based systems. Section 5 presents an overview of the advancements in bioelectrical impedance measurements in biomedical applications. Section 6 provides a discussion of the reviewed systems aimed at feature classification model extraction and final remarks for future research. Finally, Section 7 concludes this paper.

## 2. Lock-In Amplifier Fundamentals

Mainly, in lock-in detection, a reference signal of the same frequency as the measured signal of interest is needed. The reference signal may be generated in the LIA or given as an external input. A phase-sensitive detector (PSD) identifies the phase difference between the input and reference signals, and phase shifting is performed to ensure that the two signals are in phase. Finally, the amplitude of the input signal that is in phase with the reference signal is obtained [16]. It is termed “lock-in” because it locks to and measures the particular frequency of the reference signal, ignoring all other signals in the input. The operating principle is that an input signal is demodulated by a synchronous reference signal to produce in-phase or out-of-phase signals through a PSD [3]. In these applications, an LIA is able to perform phase measurements that determine the entire precision of the displacement measurement [17]. In the FPGA-based design, an LIA can provide phase estimate solutions on the accuracy of one reference signal oscillator period. Thus, an advantage of using FPGA-based designs is that, starting from a fixed system clock, the dynamic range of digital-to-time converter (DTC) only needs to cover the residual phase error within one cycle of the output clock [18].

**Figure 1:**
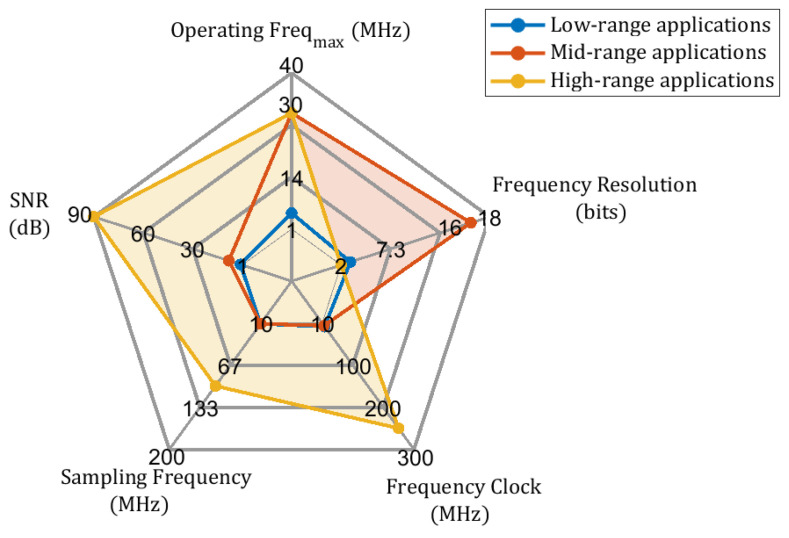
Key capabilities of revised LIA architectures: The chart considers critical parameters for the most commonly used LIA architectures, with metrics ranging from minimum to maximum values for SNR, sampling frequency, clock frequency, frequency resolution, and operational frequency.

Furthermore, correlation methods have been introduced, mainly to enhance the non-linearity detrimental effects at the reference signal. This is an approximately linear operating range for the amplifier, and it is called the linear dynamic range (LDR). Some of the main criteria for the evaluation of LIA systems that include low-noise amplifiers and mixers are the maximum power level for which inter-modulation distortion becomes unacceptable and the noise-limited operation of the amplifier. The operating range for which spurious responses are minimal is called the spurious-free dynamic range (SFDR). In [19], H. Fan et al. report a recent study that provides limited digital control to achieve a significant improvement of SFDR in smart sensor systems. Several key performance parameters, such as signal-to-noise ratio (SNR), maximum operating frequency (${\mathrm{freq}}_{\max}$), sampling frequency, clock frequency, and frequency resolution, are addressed in this paper. These capabilities, comparing both digital and analog LIAs from the current state-of-the-art, are illustrated in Figure 1 and defined in the following subsections.

The features of SFDR and SNR are used to demonstrate the trade-off between spectral leakage performance and hardware complexity, e.g., the direct digital frequency synthesizer (DDFS) design for fundamental signal generation. Similarly, the AC specifications are the most important in evaluating high-speed DAC/ADC settling time, glitch impulse area, distortion, SFDR, and SNR, since they play a critical role in the overall accuracy of the LIA system. The accuracy is a noteworthy advantage of the digital lock-in amplifiers instead of the analog ones, including the frequency synthesis, unwanted signal mitigation, and phase-sensitive detection [20,21,22]. The aforementioned approach to the DLIA structure has been used in a variety of real-time applications such as medical applications, optical spectroscopy, measuring multiple modulated frequency signals, electrical and electronic applications, and more [23]. The key capabilities outlined in Figure 1 summarize the revised LIA’s designs analyzed in this manuscript. The chart highlights critical parameters for the FPGA-based architecture, including metrics such as signal-to-noise ratio (SNR), maximum sampling frequency, maximum clock frequency, frequency resolution tailored to specific applications, and the operational frequency to low-, mid-, and high-range applications.

### Lock-In Amplifier Classification

Addressing the review of LIA architectures is not an easy task, due to the broad range of approaches in their implementation: analog vs. digital, and within digital, based on DSPs, FPGAs, multicore controllers, etc. However, the type of system suited for LIAs can be classified based on the number of mixers used, the architecture incorporated, and the specific application [24]. Lock-in amplifiers in conjunction with PSD are used in instrumentation for their capability of detecting low-amplitude signals affected by interference or noise. FPGA, digital signal processors (DSPs), and microcontroller (MCU) technologies, among others, can help in rapid prototyping and the implementation of low-cost embedded systems while obtaining both high reliability and accuracy. Owing largely to the advancements in modern FPGA devices, which can provide highly integrated hardware resources, digital block features of complex DLIA architectures can be implemented in the same device, resulting in a compact and low-cost acquisition system that is well suited for applications requiring a large number of measurement channels [25,26]. Depending on the PSD method, the lock-in amplifier can be classified based on the architecture [27]. Here, we consider two classes as follows:

1. Single-phase instrument: It has a single-PSD branch and a single reference signal. The method multiplies the excited signal by the lock-in reference using a PSD mixer or detector. Then, the PSD output is simply the product of two sine waves, and thus, the result is able to filter the removed unwanted AC signals.
2. Dual-phase instrument: It uses two PSD blocks along with two reference signals, one being phase-shifted ninety degrees with respect to the other. It is to be noted that with this configuration, the LIAs are capable of measuring both the in-phase and quadrature components. These components can greatly help in signal measuring and extraction in Cartesian or polar representations.

## 3. Single- and Dual-Phase Lock-In Amplifiers

The single-phase LIA operation is based on the frequency mixing, and a reference signal is used to single out the component of the input signal at the reference phase and frequency with known amplitudes. Thus, it can easily calculate the phase difference between these; otherwise, it may not be feasible to eliminate the AC component at the mixer output.

### 3.1. Basic Principle and Architecture

**Figure 2:**
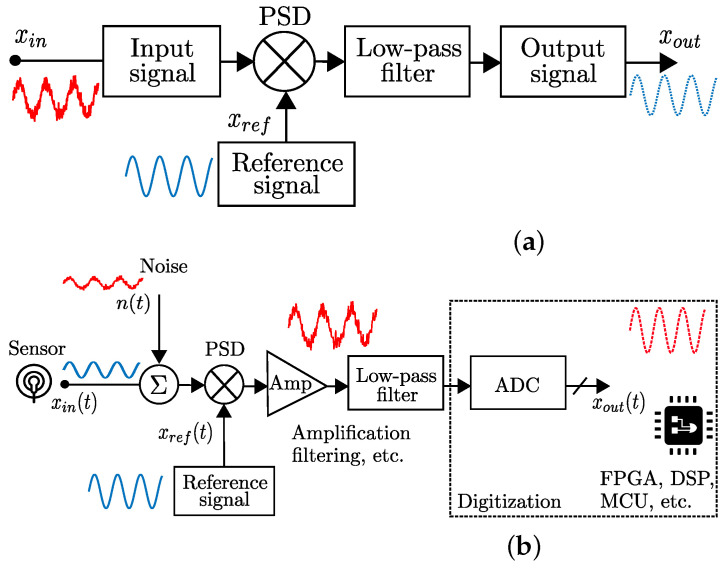
Block diagram of the single-phase LIA: (**a**) overall system, (**b**) conceptual LIA process at the system level.

The general design of the lock-in amplifier could be represented at the system level with five building blocks, as shown in Figure 2. One can see the functional diagram block from the illustration, in which the basic architecture is compounded by (i) the stimulus input signal $x_{in}$, (ii) a reference signal $x_{ref}$, (iii) a phase-sensitive detector, (iv) a low-pass filter (LPF), and (v) output signal conversion $x_{out}$. Depending on the intended application and the technology applied, each element can be a custom-hardware, cost-effective, high-performance, and optimized solution. The reference signal $x_{ref}$ will lock in to the input signal $x_{in}$, which usually comes from a sensor, a circuit previously implemented, a device under test (DUT), or an external input system. The phase-sensitive detector is a circuit that takes two signals at its input and produces an output that is the product of both signals, usually employing a mixer or multiplier. For certain sensor applications, dual-phase LIAs are demonstrated to be best suit, because it is not always possible to produce reference signals with specific frequencies. In [26], Masciotti et al. proposed the analysis extended for multi-frequency that can be used to identify the limit (if any) to improve the noise immunity by increasing the sampling frequency.

### 3.2. Dual-Phase Lock-In Amplifier

**Figure 3:**
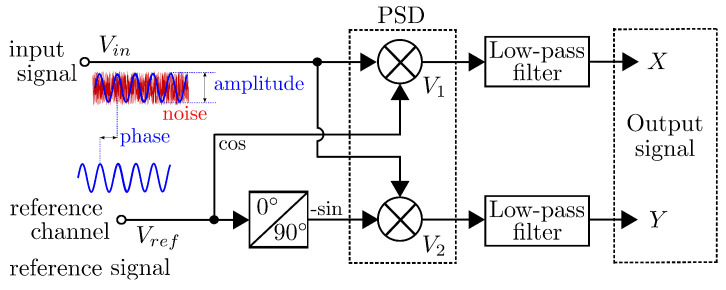
Block diagram of a basic dual-phase LIA design.

The dual-phase method for LIAs is capable of measuring both in-phase and quadrature components by using the mixing principle along with two reference signals, one being $90^{\circ }$ shifted from the other. The dual-phase LIAs can obtain the amplitude and phase of the mixing frequency signals [28]. For sensor applications, dual-phase LIAs seem to be better, because sensors are deployed in real-life scenarios, and in some cases they will be prone to severe noise, for example, atmospheric disturbances in weather sensors. However, it is not always possible to produce matched frequency signals [24]. A LIA provides an extremely narrow band-pass filter, which, at the same time, does not suffer from 1/f noise when amplifying. Figure 3 shows a dual-phase block system based on the principle of an orthogonal signal decomposition. Let us consider the amplitude and phase components of an input $V_{in}$, as shown in Figure 3, and a reference signal $V_{ref}$, to derive the PSD given as

(1) $$V_{1}=V_{in}\cdot \sin\left(\omega_{in}+\theta_{in}\right)\cdot V_{ref}\cdot \sin\left(\omega_{ref}\right),$$

where $\omega_{in}$ is the input signal frequency, and $\omega_{ref}$ is the reference signal frequency. According to the trigonometric identity $\sin\left(x\right)\cdot \sin\left(y\right)=\frac{1}{2}\left[\cos\left(x-y\right)-\cos\left(x+y\right)\right]$, Equation (1) becomes

$$V_{1}=\frac{V_{in}\cdot V_{ref}}{2}\left[\cos\left(\omega_{in}-\omega_{ref}+\theta_{in}\right)-\cos\left(\omega_{in}+\omega_{ref}+\theta_{in}\right)\right],$$

Then, the signals are applied to a low-pass filter to further signal conditioning, so higher-frequency components are neglected. Thus, the *X* is obtained as follows:

(2) $$X=\frac{V_{in}\cdot V_{ref}}{2}\cos\left(\omega_{in}-\omega_{ref}+\theta_{in}\right),$$

Similarly, in the other branch of the system, we obtain

(3) $$V_{2}=V_{in}\cdot \sin\left(\omega_{in}+\theta_{in}\right)\cdot V_{ref}\cdot \cos\left(\omega_{ref}\right),$$

and using the trigonometric identity sin(x)$\cdot \cos\left(y\right)=\frac{1}{2}$$\left[\sin(x-y)+\sin(x+y)\right]$, we can easily modify the Equation (3), to obtain

(4) $$V_{2}=\frac{V_{in}\cdot V_{ref}}{2}\left[\sin\left(\omega_{in}-\omega_{ref}+\theta_{in}\right)+\sin(\omega_{in}+\omega_{ref}+\theta_{in}\right)],$$

The signals are applied to a low-pass filter, so higher-frequency components are neglected. Obtaining *Y* as

(5) $$Y=\frac{V_{in}\cdot V_{ref}}{2}\sin\left(\omega_{in}-\omega_{ref}+\theta_{in}\right),$$

In theory, it is therefore sufficient to determine the expected value (relevant value to be measured), with the values of *X* and *Y*. The magnitude *A* is calculated as $A\left(t\right)=\sqrt{X{\left(t\right)}^{2}+Y{\left(t\right)}^{2}}$ and the phase, tan(φ)=Y/X, given by

(6) $$\varphi \left(t\right)=\arctan\left(Y(t)/X(t)\right),$$

where φ(t), is the phase that can be easily extracted from the DUT.

## 4. Digital Assessments: On the FPGA Critical Design Methodology for DLIAs

FPGAs play a critical role in addressing the leverage of modern signal processing algorithms to enhance frequency resolution, improve phase accuracy, and expand operational bandwidths in DLIAs by providing a customization platform for implementing tailored digital LIA architectures. Their capability to integrate direct digital synthesizers (DDS) for precise frequency generation, adaptive filters for noise reduction, and phase-sensitive detection algorithms ensures high-performance operation. Moreover, FPGAs allow for efficient resource utilization, enabling the realization of single- and dual-phase LIA systems with minimal hardware overhead while maintaining real-time processing capabilities. Since digital LIA platform performance needs to satisfy precise signal conditioning requirements, some numerical precision degree is mandatory. Thus, adequate methods for frequency stability, phase-sensitive multiplication, and filtering are also required. A digital approach can jointly provide a computationally much simpler implementation than an analog one such as in [29], which also yields a lower error estimation of the underlying signal amplitude. Furthermore, when designing LIAs in an analog implementation, some errors are difficult to mitigate due to the inherent characteristic of analog electronics, whereas in digital implementations, one can mitigate some of those error sources by modifying digital functional blocks or applying signal enhancement algorithms, thus improving accuracy. The FPGA-based digital LIA promises useful improvements for weak signal detection technology, such as those needed in the fields of electronic science, signal processing, and sensor technologies [30,31,32]. FPGAs are flexible and robust devices for this purpose, which, in addition to VHDL hardware description language, allow describing through behavior models by the register-transfer level (RTL) and structure of digital components, such as precise DDS, which is a key component for ASIC design and implementation. For example, in sensors and measurement systems, finite precision controllability for the signal extraction is accomplished, thus reducing some error-phase measurement system imperfections [33,34]. In turn, intrinsic signal noise introduced by detectors, filters, and amplifiers features certain levels of unavoidable noise, and some can be eliminated by improving the generator and/or detector design. We summarize some of the critical reliability requirements with regard to the digital inaccuracy; these features are salient:

- DDS resolution (spectral purity DAC/ADC enhances).
- Low complexity in arithmetic logic (multiplications).
- Signal/noise power contributions (DDS-to-DAC).
- Clock synchronization to uncertainty reduction.
- Total harmonic distortion (mixing process) [35].

**Figure 4:**
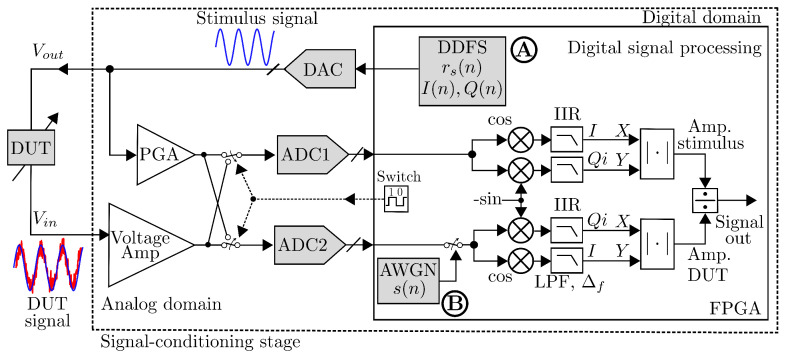
DLIA block diagram with main modules.

An FPGA-based LIA design enables flexible hardware adjustments in the field, resulting in significant cost savings. Note that in the proposed techniques, critical performance is dependent on the reference signal, which becomes of relevant for high precision. Figure 4 sketches a dual-phase DLIA platform with the main modules implemented in FPGA hardware. Thus, DDFS is a key component, especially in instrumentation and measurement, which becomes a functional part for high-precision systems (see [25,36,37,38,39]), which is crucial in both single- and dual-phase DLIAs architectures. In DDFS designs, the effect of finite precision on digital sinusoidal frequency synthesizers manifests itself as spurs in the spectral representation of the output sine wave. These effects are directly related to the phase angle precision limitations since the derived phase of the DDFS digital oscillator tends to be periodic in time and to contribute to the generation of harmonics and spurious. These drawbacks can be reduced by considering the following:

- Phase noise in DDS. In digital LIAs, the signal reference needs to determine DDS periodicity accordingly in order to appropriately reduce the phase noise at these frequencies. This can be accomplished by introducing a random signal of suitable variance into the derived phase, thus facilitating the reduction in the likelihood of identical values over time.
- SFDR strength ratio specifications. Spur-reduction techniques state that adding noise into the data path raises the overall noise level within the oscillator, which tends to reduce the noise localization and can provide significant SFDR enhancement.
- Frequency-phase likelihood in DDS. The requirements to reduce spur levels are dependent on many factors. The likelihood of repetition of derived phase values and resulting spurs, for a given angular precision, are closely linked to the ratio of the sampling frequency to the desired output frequency from any DDS source. An integral ratio clearly results in high-level spurious frequencies, while an irrational relationship is less likely to result in highly correlated noise at harmonic frequencies.

### 4.1. FPGA-Based Lock-In Amplifier System Signal Enhancement

It is well known that a digital LIA structure uses a synthesizer DDS-to-DAC output. DDS contains a digital block called a phase accumulator of $2^{n}$-bit samples, which produces the digital sinusoidal signal. A large word size is needed to produce the low-hertz step size. Ultimately, the phase accumulator outputs to a sine look-up table (LUT). The large word size of the phase accumulator means that the word output to the DDS must be truncated to fit the limits of the DAC’s input. This truncation leads to small discontinuities in the generated pattern, creating greater harmonic distortion [35]. These potential issues unavoidably cause deterministic timing errors, often referred to as deterministic jitter, that appear in the output spectrum as spurious tones. A preferred solution is using dither seed in the generation of the phase accumulator output, which can reduce these “truncation spurs”. Fortunately, these imperfections can be mitigated significantly with an efficient spread spectrum technique for a near-optimal spur-reduction performance. To extract enough useful information from the low signal-to noise ratio (SNR) signals, supposing a periodic waveform signal measured as in Equation (7), and, according to a given variance, modeled as the additive Gaussian, the white noise is:

(7) x(t)=A·sin(ω(t)+ϕ)+n(t)

(8) $$\mathrm{SNR}=10\log_{10}\left(\frac{A^{2}}{2\sigma^{2}}\right)$$

As the approach of this work, we have concentrated on signal enhancement awareness to provide a digital signal conditioning stage as a key design consideration in the signal reference generation module of a typical DLIA. Notice that in the present work [40], the reference signal frequency resolution of 0.0291 Hz is ensured (calculated as $f_{min}$ = $f_{s}$/$2^{32}$), and for specific bioelectrical signals under 10 KHz, this is more than enough for these applications. Please also note that in terms of the dynamic reserve (DR) and SFDR, those metrics have a strong relationship to the accuracy of measurement in the capabilities of any LIA system, and can be calculated as follows:

(9) $$\mathrm{DR}=20\log_{10}V_{d}/V_{s}$$

where $V_{d}$ is the amplitude of the disturbance and $V_{s}$ is the amplitude of the signal of interest.

#### 4.1.1. FPGA-Based Lock-In Detection for Multi-Channel Chemical Species Tomography

**Figure 5:**
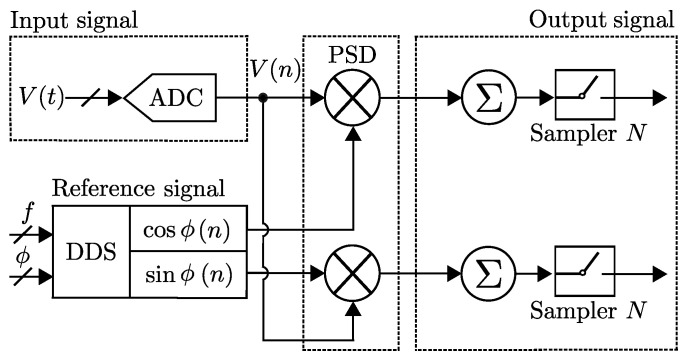
Block diagram of an FPGA-based LIA for multi-channel chemical species tomography.

A DLIA is based on a quadrature demodulation, by which both the amplitude and phase of the signal can be obtained [25]. The system is implemented using a Nexys 3 development board with an FPGA Xilinx Spartan-6. The LIA reference signals were tuned at 50 kHz. These results validate the feasibility of the proposed system to exploit the properties of FPGA platforms to implement low-profile, low-weight, and low-cost DLIAs. Figure 5 shows the design of the dual-phase lock-in amplifier. The input signal is then converted from analog to digital, and the two reference signals of the same amplitude are used, at the same angular frequency ω, but with $90^{\circ }$ offset relative to each other; this to be able to obtain the signal amplitude and phase. By multiplying the input signal with amplitude *A*, with the two reference signals with amplitude *B*, we obtain the in-phase and quadrature components.

#### 4.1.2. Lock-In Amplifier for Atomic Force Microscopy Systems

In [39], a new efficient method for implementing the multichannel digital lock-in technique is presented, which is able to measure the amplitude and phase of multiple modulated frequency signals to solve the two most important problems in atomic force microscopy (AFM) systems, which are resolution and cost. Nonlinear contact between the tip and sample in tapping-mode AFM systems induces higher harmonics, which may be useful for the extraction of some characteristics of the sample [39]. The FPGA Xilinx Spartan-3 results demonstrate that the proposed architecture is superior to previous structures, especially in the hardware area and power consumption. The design is very similar to the basic dual-phase LIA system, where the reference signal comes from a DDS component on FPGA Xilinx Spartan-3. The input signal in this design is formed by the signal coming from the device under test (DUT), because it is a digital system, and the DUT may be an analog system. To measure the amplitude response of a nonlinear system, the probe should initially be excited by the sinusoidal signal. In a digital LIA, this signal can be realized by the DDS on FPGA and a DAC to convert it into analog form. It is worth mentioning that microscopy is one of the most popular applications of LIAs, used in experimental physics applications to design instruments that measure signals that are affected by a lot of noise [41,42].

#### 4.1.3. FPGA-Based LIA with Sub-ppm Resolution

**Figure 6:**
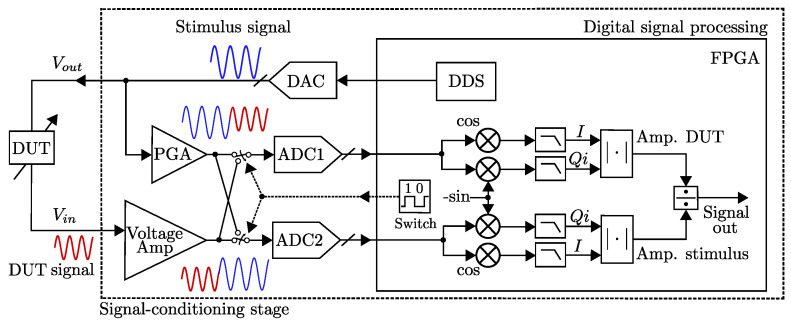
Block diagram of an FPGA-based LIA with sub-ppm resolution working up to 6 MHz [38].

In [38], Gervasoni et al. reported a synchronous phase-sensitive architecture for sub-ppm resolution measurement system as shown in Figure 6. Here, the two branches are driven by a switch to sampling simultaneously at {ADC1, ADC2}, respectively. Note that gain fluctuations are canceled out by means of a ratio operation between the two amplitudes. The signals are reconstructed in real time using an FPGA to obtain their discrete-time sampled data as x(n)=I(n)+Qi(n) synchronously established by a PLL. The two signal amplitudes (and phases) are calculated with a dual-phase demodulation as in a standard LIA. The realized enhanced-LIA (ELIA) instrument comprises a generation channel, two acquisition channels, and an FPGA, and the measurement frequency can be up to 6 MHz. The design realization is based on a Spartan-6 FPGA from Xilinx (mounted on a module Opal Kelly XEM6010 that includes a PLL, external memory, and USB interface). A PGA provides an attenuation/gain in the range of (−22 to 20 dB). The realized enhanced LIA (ELIA) is a high-resolution replacement of standard LIAs, for example, in sensors or in device characterization applications, without requiring changes in the experimental setup or calibration.

### 4.2. Low-Cost Accurate Phase Measurement System

**Figure 7:**
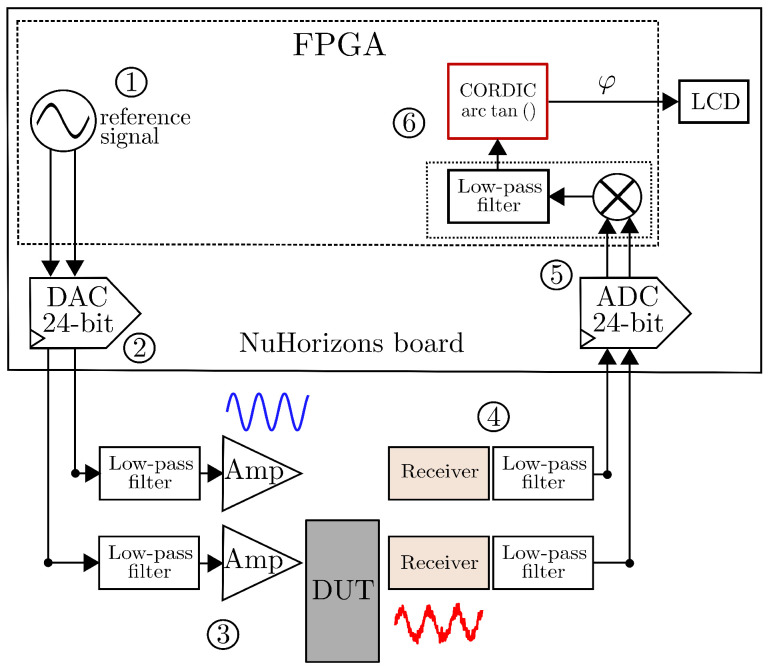
FPGA-based design of a digital phase measurement system [36].

In [36], Vandenbussche et al. reported a low-cost accurate phase measurement system based on a DLIA. In particular, we consider the structure with six stages depicted in Figure 7 for a conventional FPGA-based DLIA implementation. The blocks 1 and 6 depict the design built using an FPGA Xilinx Spartan 3A-DSP as a pre- and post-processing circuit to sending capture data. For the blocks 2 and 5, the system employs a NuHorizons Spartan 3A-DSP kit NH-SPAR3ADSP-EVL with a Maxim MAX11040K DAC/ADC to provide an adequate sampling procedure. The overall digital design is implemented in the FPGA NuHorizons kit, and a Xilinx Spartan 3A-DSP XC3SD1800A with an FPGA clock sampling at 80 MHz. At the blocks 1 and 6, the circuit also ensures a clock frequency that is divided by ten inside the FPGA and supplied to the Xilinx DDS IP Core using an 8 MHz reference clock. As noted in block 1; the reference signal is generated with a DDS component using a parameterized IP-block able to generate a cosine/sine signal of equal frequency. Both channels are converted from digital to analog by the DAC.

The cosine signal, which is the input signal, passes the DAC reconstruction low-pass filter, the driver circuit, and the DUT. The reference signal passes a DAC reconstruction filter and driver circuit. For the blocks 3, 4, and 5, the input and the signal from the reference channel are both anti-aliasing filtered and simultaneously sampled by the ADC and sent back to the FPGA. Similarly, in blocks 5 and 6; the two signals, composed of the input signal and the reference signal, are sampled simultaneously and multiplied with each other in the PSD and low-pass-filtered. In the block 6, the product output is subsequently digitally low-pass-filtered. Also, the arc-tan function computes the associated angle difference by using a Xilinx parametric CORDIC IP φ information at the output signal. Blocks 1 and 2 address the frequency resolution constrained by the hardware DAC’s 24-bit limitations, resulting in an output SNR of approximately 6 dB per/bit, corresponding to a spurious-free dynamic range (SFDR) of around 96 dB/Hz.

## 5. Bioelectrical Impedance Measurement

Disregarding the method or architecture used to perform the measurement of the signal properties of different systems, there is no doubt that biomedical and healthcare applications of biological signal measurement have been responsible for continuously pushing the electronic hardware to their limits of performance and miniaturization. The measurement of the electrical bio-impedance (EBI) of the human body and its organs, tissues, and cells, better known as bioelectrical impedance analysis (BIA), has been widely used in medicine because of the relative simplicity of its technical implementation, its feature of being non-invasive, relatively inexpensive, and its property of performing in almost any subject because of its portability and miniaturization capabilities. These characteristics make this technique the most suitable method for monitoring the state of tissues and organs, both in vitro and in vivo, and it has great potential for the development of new applications.

EBI analyzers with acceptable accuracy, reliable measurements, and compact hardware implementations are needed for the most relevant clinical applications, for instance, to be suitable for on-chip realization in implantable devices [43,44,45]. A technique called Electric Cell-Substrate Impedance Sensing (ECIS) provides label-free and real-time detection of cells, which is emerging as an alternative or assistive method to traditional biochemical assays for diagnostic and pharmaceutical applications [46]. Its compatibility with a liquid environment combined with low cost and reduced size, with respect to optical techniques, makes bioimpedance measurements one of the most promising transducer mechanisms for lab-on-a-chip and biochip platforms [47]. This sensing methodology has been applied to different biomedical applications, such as cell growth monitoring, impedance-based flow cytometry [48], and impedimetric affinity biosensors, the latter being one of the most promising tools for point-of-care diagnostics [47]. The EBI analysis is widely used to quantify fat-free mass (FFM), body fat (BF), body cell mass (BCM), total body water (TBW), extracellular water (ECW), and intracellular water (ICW) in healthy and ill subjects [49]. It can be also used to perform several studies on body bigger parts (e.g., rheography and plethysmography) [50], the examination of particular organs, glands, or parts of the body (e.g., heart, liver, larynx, prostate, breast, blood, etc.), the examination of some selected fragments of tissues, and measurements of single-cell impedance [44,51]. More recently, it was also shown that EBI analysis has sufficient sensitivity to replace reference methods for the assessment of body composition in athletes [52], and it has great potential to be used to observe the proper body development in children and adolescents [53] and to predict risk for gestational diabetes mellitus by measuring maternal body composition [54], just to mention a few high-impact applications in the biomedical and health industry.

Electrical impedance spectroscopy (EIS) applied to biological tissues, also known as bioimpedance spectroscopy, is a powerful and versatile technique used to study the frequency response of the electrical properties of biological materials noninvasively. In this test, a low-amplitude electrical signal is injected into the tissue sample or body parts to characterize the sample in terms of its bioimpedance. Since biological tissues are developed with biological cells, which exhibit complex electrical responses, under an alternating electrical excitation, the bioimpedance varies with the tissue anatomy and composition, and the applied signal frequency modifies the current penetration and conduction paths [55]. The variation of bioimpedance as a function of frequency is a valuable source of information about an examined tissue. This technique has been widely used to assess the condition of organic tissues in vivo, in vitro, and ex vivo and for various applications in many areas of research and clinical practice [56,57,58,59,60].

For instance, bioimpedance measurement using EIS has been applied to differentiate cancerous tissues in a variety of organs, including breast, cervix, skin, bladder, and prostate [60], to assess skin hydration, to detect breast cancer, to measure fluid volumes on limbs, for respiratory monitoring [61], to detect subjects with cardiovascular disease risk factors, and to improve the prediction accuracy for measuring abdominal fat distribution using machine-learning-based algorithms [62]. Bioimpedance measurement in tissue is one of the parameters that allow ischemia monitoring in living bodies [63]. This measurement was also used to show the influence of skin impedance on biological potential measurement, to measure skin moisturization using skin admittance, and to analyze gait analysis using lower-leg electrical impedance [64]. Many biomedical, immunological, and pharmaceutical studies require highly homogeneous populations of biological particles separated from heterogeneous mixtures such as peripheral blood or even clonal cell lines with differing characteristics. Impedance-activated microseparation is a very sensitive technique, which can size, count, and isolate particles based on the bioimpedance measurement [65].

Thus, the understanding of the electrical current conduction in biological tissues and the development of new methodologies for the quantification of this phenomenon are of great relevance to improve techniques like bioelectrical impedance analysis (BIA) and electrical/electrochemical impedance spectroscopy (EIS).

### Computational Prediction: A Robust Machine Learning Approach

**Figure 8:**
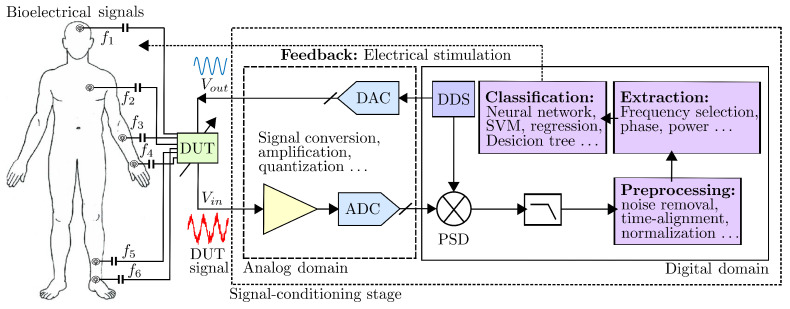
Block diagram classification with analog components and digital FPGA-based design blocks for the analysis and digital conditioning of bioelectrical signals.

**Figure 9:**
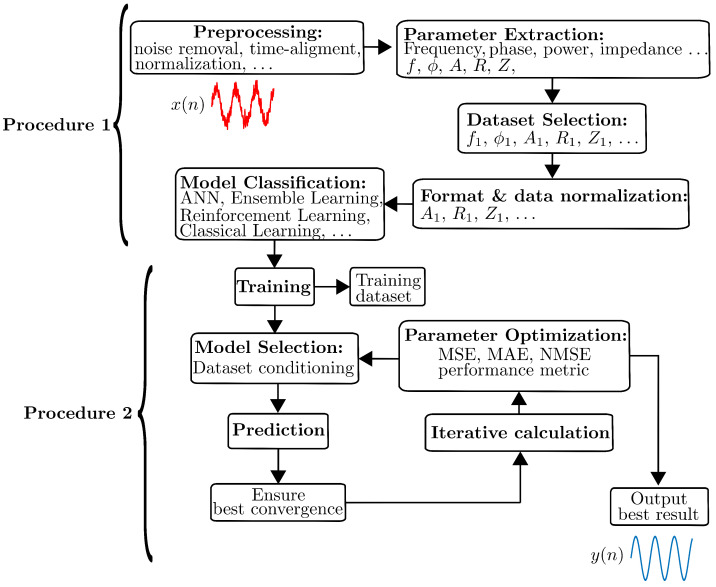
Block diagram of the procedure for signal extraction, selection, and model classification for feature optimization.

The underlying embedded technology aims to make optimal use of hardware realizations to address more complex and flexible circuit architectures that can be applied to improve the digital signal acquisition and conditioning performance with practical limitations. This notion is faced by DSP algorithms for implementing the complex processing of biological systems acquired from reading sensor signals that present intrinsic properties with highly irregular, non-stationary, and heterogeneous morphologies. This complexity implies the necessity of suitable methods, such as the supervised and unsupervised classification of machine learning (ML) algorithms, which contribute to providing several promising results to modern medicine devices [66]. The goal of the classification algorithm is to distinguish different signal states accurately [14]. Thus, the performance of classification algorithms can help to acquire high-quality signals to characterize true and false positives to provide further metrics and figures of merit such as receiver operating characteristic (ROC) curve and area under the curve (AUC) analysis. In [67], N. Dey et al. reported several models based on clustering and classification approaches such as convolutional neural networks (CNNs), decision trees, and support vector machines (SVM) that have been successfully applied to medical imaging. However, biomedical signal analysis has yet to fully benefit from this novel approach. Figure 8 shows the block diagram of the analog components and digital hardware building blocks based on FPGA-based design blocks for the analysis of bioelectrical signals with sources of multiple wire connection frequencies with channel selection [68]. The block diagram shown in Figure 9 denotes the two procedures associated with an automated process for the extraction of the main features in a given adaptive processing and classification of sensor signals, following a step-by-step parameterization. The model classification provided in procedure 1 in Figure 9 integrates various techniques, including ensemble learning approaches and artificial neural networks (ANNs), serving as examples for feature extraction. Additionally, parameter optimization is performed using metrics such as the mean squared error (MSE), mean absolute error (MAE), normalized mean squared error (NMSE), and the coefficient of determination. These metrics evaluate the model’s performance in regression analysis and assess its effectiveness in minimizing prediction errors.

In [69], a system is reported to determine the optimal biosignal characteristics from recorded sampled data points over time periods for the detection of driving stress from electrocardiogram (ECG) signals. An extensive review is addressed in [70] to compare the accuracy of classification, implementation complexity, invasiveness, and targeted application for electromyography (EMG), electroencephalogram (EEG), and rapid eye movement (REM). An approach guided by learning paradigms can successfully aid in the development of advanced healthcare diagnostic systems for biosignal analysis [71].

**Figure 10:**
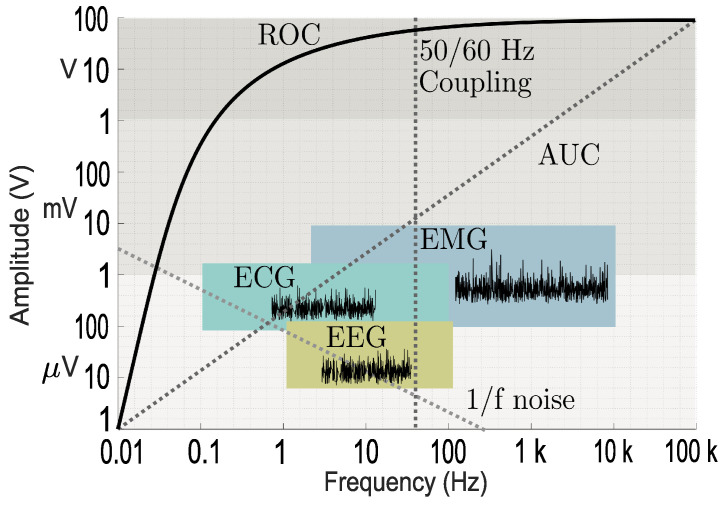
Signal characteristics with the frequency/amplitude relationship.

**Figure 11:**
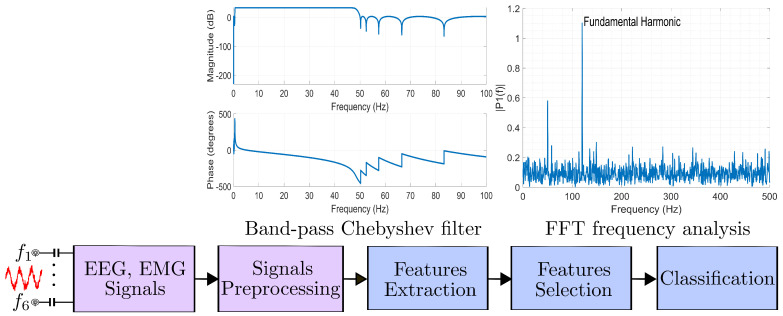
System key building blocks for the processing signals at the hardware integrated circuit.

Figure 10 shows different types of signals with typical frequency, amplitude, and 1/f noise relationship levels. Figure 11 summarizes the implementation building blocks to continuously sample and process the various types of bioelectrical signals from electrodes shown in Figure 8. The concept of developing the architecture in the digital domain enables us to reach an adequate algorithmic and digital processing circuits, which in turn analyze and classify the stream of digital signals using well-suited pre-defined algorithms to provide dedicated FPGA/ASIC hardware. The AUC classification captures key signal characteristics in the time domain, which can be transformed into frequency representations via a fast Fourier transform. Calculating the AUC of the power spectral density quantifies the energy distribution of EEG, EMG, and ECG signals across frequency bands [72], offering insights into signal power and facilitating the analysis of physiological or pathological processes within specific frequency ranges, as illustrated in Figure 10.

## 6. Discussion

We have introduced a comprehensive summary of applications, including industrial electronics [73], telecommunications, and healthcare biomedical circuits [12,21,43,44,66,69,74]. The design implementation for these applications has attracted the attention of the industry and the scientific community, starting from a few basic assumptions on the design [29], analysis [26,68], measurement, and modeling techniques which are important to address rigorously engineered LIA systems [34]. Moreover, a key lack of these architectures in voltage resolution control for the detectable instrument has to be fixed by an input range for both the single and dual-phase and is independent of the input signal amplitude [38]. Note, however, that the dual-phase LIA usually deals with an unavoidable and intrinsic 1/f flicker noise of the reference voltage (either used by the DAC and ADC), and most applications result in signal amplitude modulation [17,24,26,38,39,75]. LIAs are strongly affected by cyclostationary effects that mainly originate from circuits such as the ADC and DAC, which are also correlated with conversion gains and fluctuations. In [38] a standard implementation is demonstrated to provide benefits in sub-ppm measurements, i.e., filtering bandwidth of 1 Hz to switching frequency greater than 1 kHz so that the resolution will be flat at sub-ppm values. However, by lowering the switching frequency [21,76], the resolution suffers from spectral degradation due to the overlapping with the side harmonics of the 1/f noise components. Then signal-conditioning measurements become dependent on the frequency of the input’s spectral density, which can be compensated with an operation where a finite frequency response exists. Furthermore, some noise effects on digital LIA architectures can be scaled or shifted using convolution [75], which helps to keep SNR at maximum levels. In this context, it is well known that lower phase noise is achieved with a larger size or by paralleling several devices. Thus, FPGA digital components that operate with main clock synchronization, (i.e., if a frequency synthesis is dependent on clock-aware routability, some issues such as the sampling clock jitter can be removed given the time base) are an overall cost [34,77]. The convenience of the digital alternatives is that it benefits the accuracy of detecting quantitative noise contributions.

The assumption behind the methods of quantization errors and resolution in signal generation lie in the achievable digital LIAs performance, which shares a strong relationship with the optimization methods, such as in terms of numerical precision problems. To the extent of our knowledge, in [26] Masciotti et al. introduced a great advance in the analysis of a digital lock-in technique for practical noise reduction and discrimination, where the design can detect multiple signals at different modulation frequencies. Consider that for the enabling technologies that involve medical applications comprising several IoTs providing smart healthcare devices into one SoC, measurements present fundamental limits that are related to the lock-in amplifier relative resolution. Similarly, it should not be ignored that practical sensor-stimuli systems (EEG, gas, audio, etc.) have lagged far behind the application flexibility given their steady-state discrete-time conditions [76]. Despite the capabilities provided for analog lock-in amplifiers, a few examples from the literature of proof-of-concept experiment design CMOS technologies provide flexible functionalities or enable interoperability between the measurement system and the sensor. In other words, the supporting process of signal extraction is in most cases limited to specific technical restrictions related to the signal conditioning stage, extraction, and the measurement process [21,76,78].

### 6.1. Challenges and Future Directions for Driven Machine Learning Architectures

Bioelectrical impedance sensors are pivotal tools in the domain of health monitoring, providing valuable insights into various physiological parameters. The review concludes by addressing the challenges encountered in employing machine learning algorithms for feature extraction from bioelectrical impedance sensors. The extraction of meaningful features from impedance data is a critical step in enhancing the accuracy and reliability of health-related predictions. Additionally, potential future directions, including the integration of deep learning techniques and real-time applications, are outlined to provide a roadmap for further research in this domain. The incorporation of AUC analysis enhances the precision of feature extraction processes, thus contributing significantly to the advancement of health monitoring technologies. Emphasis is placed on algorithms’ ability to exploit the enhanced impedance signals obtained through lock-in amplifiers, as explained in the procedure extraction and the model feature classification in Figure 9. Thus, this review provides some key comparative metrics such as accuracy, sensitivity, and specificity to quantify their performance. The development of compact instrumentation and the measurement and analyses of the observed data use multiple stochastic and machine learning techniques to bring out the best correlation fit between the glucose concentration and a specific feature of the electrical signal ML techniques applied to the extraction of data from LIAs [79] to provide pioneers with compact photoacoustic spectroscopy systems with a lock-in amplifier as basis circuit that also can integrated with machine learning, marking a significant step toward wearable glucose monitoring devices.

### 6.2. Comparative Table: A Systematic Review

Some practical considerations of the state of the art for LIA architectures are summarized in Table 1. The discussion in Section 4 was devoted to detailing the applications available for a wide range of frequencies. In the same way, some underlying aspects of LIA technology such as the design, application, and signal reference generation were addressed. In the literature, Chighine et al. [25], Vandenbussche et al. [36], and Ayat et al. [39], proposed some FPGA-based architectures where an overall LIA system is conceived. The most relevant features ofn the FPGA devices are in the requirements and reliability of signal conditioning aspects, which are crucial for these specific sensor applications. In most cases, the digital DDS architecture [16,18,26,34,37,77] requires a precise tuning frequency control, which sometimes makes critical to apply a sampling rate for an equivalent sampling frequency. Table 1 and Table 2 present an overview of works that attain a good synchronization and frequency resolution for each platform/device design for several LIAs systems. As an example, such a design principle is to ensure the Nyquist criterion, and the optimal detection process is simple and can ensure a reliable and straightforward characterization if the maximum output frequency is provided on a factor of 1/3 for a sampling frequency [38,40]. These limitations on critical sensor applications tend to result in inaccurate sub-Hertz frequencies at the extraction method and thus do not provide a precise normalized amplitude at the stimulus signal [34,38]. Recent developments present attractive solutions to address the digital precision of LIA system-level requirements. In [80], Pfeiffer et al. proposed a module for biomedical health issues over the terahertz range to constitute a sequential system implementation with clocking control and a lock-in amplifier-based digital readout mode. Likewise, the implementation of silicon technology, such as the approach using DSP, has advanced to the point of providing special signal conditioning functions on LIAs, thus enabling high-precision measurements on sensor systems through FPGA devices [16,25,26,33,34,36,38]. In [36], Vandenbussche et al. proposed a practical investigation to calculate the causes of inaccuracy from a system derivation that considers aspects like linearity, quantization effects [18], differentiation, logarithmic and anti-logarithmic conversion, and peak-to-peak and phase-sensitive detection [11,33,34,36], in which signal quality improvements are closely related to phenomena, such as cross-sensitivity, non-linearity [19], and unwanted signals. In [19], Fan et al. proposed a technique to detect physical, chemical, or biological quantities in sensors by optimizing SFDR from the ADC signal extraction to smart sensor systems, which can enhance the inaccuracy of the delayed measured phase for conventional LIA architectures [19,33,81]. On the other hand, the knowledge of the experimental designs in which non-idealities for synthesizing frequency limits for the signal recovery process are strongly dependent on systematic errors due to harmonics [43]. In this context a study with the reference frequencies is validated in [11] and for the designs reported in Table 1. Another aspect that occurs with successive measurements is related to phase distortion that is highly dominant; thus, it becomes crucial to apply calibration methods to reduce the uncertainty in the experimental system [82]. Gervasoni et al. [38] experimentally demonstrated an LIA system based on a dual-channel ADCs full-duplex, which can acquire the signal from the DUT and the stimulus signal. In this architecture, the LIA enhancements allow the compensation of the slow gain fluctuations of both the DAC and ADC, which can considerably reduce phase mismatch effects at the extraction chain. In [83], Sarma et al. presented an analysis of theoretical SFDR resolutions ≥ 12-bit ADCs. A key aspect is related to the convenience of the DDS resolution accuracy for the designs summarized in Table 2, which also determines precision in the output signal. Such a key condition is difficult to fulfill in the case of high-frequency digital LIAs, with sampling rates of tens or hundreds of MS/s.

### 6.3. Final Remarks and Future Research

The overview of LIAs in this paper emphasizes the digital design capabilities to enable the LIA instruments interfacing with sensor devices [8,10,30,31,32]. In general, some critical LIA requirements for higher-level applications that interact with a sensor, such as their topological arrangement, can be related to voltage phenomena associated with the amplification, filtering, and digitization. Most digital LIAs use a PLL to generate a stable frequency. This indicates that in the phase domain that governs the reference and input signal, a phase relation and its frequency are responsible for the progression of phase locking for both the stimuli and the reference signals [26]. Some frequency-dependent key attributes in LIAs are related to three considerations:

- Frequency-commensurate support: It is important to emphasize that for the commensurate agreement lock-in frequencies (i.e., working at precisely 10 MHz on a lock-in with 100 MHz sampling rate), a frequency with a factor of 1/10-th of the sampling rate has to be ensured [40].
- Uniformly sampled signal rational samples: In digital LIA instruments, it is often necessary to consider incommensurate frequencies [89], where the ratio of the two signal frequencies is irrational. This is an important sub-case of a commensurate frequency relationship, namely $f_{1}/f_{2}$ = n/m for the integers *n* and *m*.
- Time-invariant, phase normalization, and commensurate signals: A signal conditioning stage must be present on a quick verification of the DUT response that treats the nonlinear stimuli.

In addition to the signal conditioning and integrity techniques, some LIA systems where signal processing is compounded with correlation methods are certainly valid and sufficient to identify and count events where the SNR is already good, even if these electrical signals become noisy due to severe conditions of temperature, pressure, humidity, and drift [18,26,29]. In some LIA applications under very noisy environments, it sometimes becomes impossible to apply correlation techniques; however, digital adaptive filtering techniques can help to recover the data (see Ref. [74]). This also facilitates a more accurate estimation given the subsequent dependence at the inter-stage quantization process (e.g., DDS, DAC-ADC, filtering) [83]. Thus, for a noisy measurement environment, improving the reattained SNR (assuming a reasonable calibration procedure) of the system is crucial. In some cases, SNR reduction can modify the filtering so that any correlation method can be subsequently applied to the filtered data [26,30,34].

## 7. Conclusions

In this paper, we have provided an overview of recent digital LIA application developments. We emphasized in this investigation the context of a tailor-made real-time embedded system, where the stimulus frequency and acquisition can provide a stable operation above Hz up to tens of MHz. Thus, by knowing the capabilities of the application of signal processing techniques, lock-in amplifiers’ performance can be improved rapidly from an RTL digital design perspective. For the reviewed applications, DDS digital frequency controllability can accelerate the embedded LIA design applications, which in turn can provide an effective and efficient tradeoff between accuracy and hardware consumption. Similarly, from the digital point of view, large systematic errors in both single- and dual-phase of the described lock-in phase’s system applications tend to decrease the conditioning signal methods’ performance for most practical sensors, which in turn influence the interoperability features for the measurements and design limitations. As we highlighted, for the critical design features for DLIA’s architectures, the SFDR performance is a critical characteristic in FPGA-based design and is especially vital in applications requiring high sensitivity and specificity, such as biomedical signal processing and advanced sensor systems. Therefore, we also emphasize that the optimization of SFDR to DLIA’s designs not only enhances measurement accuracy but also increases the applicability of these systems across diverse and complex use cases. This enhancement can not only improve the calibration stage for digital dual-phase LIAs, but it can also improve linearity in the DUT signal extraction. From this perspective, an accelerated FPGA-based LIA digital architecture implementation can be improved by ensuring enhancements of spectral purity. Also, our findings from the actual needs of the far-reaching significance of frequency-dependent LIAs operation has a good compromise among resolution, control, switching, and system implementations, such as in sensors that need high specificity and sensitivity.

## Figures and Tables

**Table 1:** Comparison of DDS performance in seminal works on digital LIA systems.

Digital Architectures			
**LIA Class, References**	**Freq. clk.**	**Device/Platform**	**Key Feature: Ref. Description**
Single-phase, [36]	250 MHz	FPGA Xilinx Spartan-3	A low-cost digital LIA system to accurate phase measurement.
Single-phase, [84]	100 MHz	TI TMS320 DSP series	A low-power operation DSP for remote and battery-powered operations.
Dual-phase, [12]	250 MHz	FPGA Xilinx SoC XC7Z100	A system application for microfluidic impedance measurement
Dual-phase, [25]	100 MHz	FPGA Xilinx Spartan-6	A multichannel optical chemical tomography system.
Dual-phase, [39]	500 kHz	FPGA Xilinx Spartan-3	A digital LIA to measure the amplitude and phase of multichannel modulated frequency signals.
Dual-phase, [75]	70 kHz	Microcontroller DSP-based	A low-cost LIA, that recovers the weak signal under extremely noisy conditions.
Different approaches [38]	6 MHz	FPGA Xilinx Spartan-6	A DLIA architecture that allows to compensate low-frequency gain fluctuations added by the DAC/ADC.
**Analog Architectures: Seminal Works**			
**LIA Class, References**	**Freq. clk.**	**Technology**	**Key Feature: Ref. Description**
Single-phase, [21]	14.64 MHz	CMOS 0.35-μm	A LIA for optical sensing and spectroscopy applications.
Single-phase, [76]	10 kHz	CMOS analog multipliers	A system for electroencephalogram biomedical applications.
Single-phase, [78]	25 Hz	CMOS 0.35-μm	A detector for measurement of small, slow, and noisy signals.

**Table 2:** Comparison of DDS performance with several digital LIAs systems.

Reference	DDS Resolution [bits]	Operating Freq_max_ [Hz]	SNR [dB]
Huang et al. [12]	14-bit	30 MHz	20
Chighine et al. [25]	12-bit	50 kHz	–
Vandenbussche et al. [36]	16-bit	15.62 kHz	90
Ayat et al. [39]	14-bit	500 kHz	2
Gervasoni et al. [38]	14-bit	6 MHz	42
Proposed work, [40] ^†^	14-bit	12.5 MHz	90
Sonnaillon, et al. [85]	10-bit	5 MHz	50
Milhem et al. [76]	–	10 kHz	15
Das et al. [84]	10-bit	5 kHz	–
Rahmannuri et al. [86]	12-bit	10 kHz	15.6
Bhattacharyya et al. [75]	16-bit	5 kHz	24.09
Liu et al. [87]	16-bit	100 kHz	–
Cheng et al. [88]	12-bit	1 MHz	–

^†^ Considering the DDS signal reference with phase dithering enhancement (up-to four tones), Freq_max_ is given with a 1/10 factor for a 125 MHz clock.

## Data Availability

Not applicable.

## Abbreviations

ADC	analog-to-digital converter
AFM	Atomic force microscopy
ARM	advanced RISC machines
ASIC	application-specific integrated circuit
BPF	band-pass filter
CMOS	complementary metal-oxide-semiconductor
DAC	digital-to-analog converter
DDFS	direct digital frequency synthesizer
DDS	direct digital synthesis
DLIA	digital lock-in amplifier
DSP	digital signal processor
DTC	digital-to-time converter
DUT	device under test
FPGA	field programmable gate array
HDL	hardware description language
HPF	high-pass filter
IP	intellectual property
LDR	linear dynamic range
LFSR	linear-feedback shift register
LIA	lock-in amplifier
LNA	low-noise amplifier
LPF	low-pass filter
LUT	look-up table
MCU	microcontroller unit
NCO	numerically controlled oscillator
PGA	programmable gain amplifier
PLL	phase-locked loop
PMD	phase-modulation-demodulation
PSD	phase sensitive detection
PXI	PCI eXtensions for Instrumentation
RISC	reduced instruction set computer
RMS	root mean square
RTL	register-transfer level
SFDR	spurious-free dynamic range
SINAD	signal-to-noise-and-distortion
SNR	signal-to-noise ratio
SoC	system on a chip
THD	total harmonic distortion
VHDL	VHSIC hardware description language
IoT	internet of things
